# Home and Neighborhood Food Environment Measures and Weight-for-Age z-Scores Among Children Aged 1–5 Years: An Exploratory Cross-Sectional Analysis

**DOI:** 10.3390/nu18142352

**Published:** 2026-07-17

**Authors:** Alanis C. Stansberry, Camille R. Schneider-Worthington, Sarah-Jeanne Salvy, Demetria R. Pizano, Suzanne Judd, Keith Pearson, Megan Word, Gareth Dutton

**Affiliations:** 1 Department of Nutrition Sciences, University of Alabama at Birmingham, 1675 University Blvd, Birmingham, AL 35233, USA; 2 Division of General Internal Medicine and Population Science, Department of Medicine, University of Alabama at Birmingham, 1720 2nd Avenue South, Birmingham, AL 35294, USA; 3 Department of Medicine, Research Cedars-Sinai Medical Center, 700 N. San Vicente Blvd., West Hollywood, CA 90069, USA; 4 Department of Psychology, University of Alabama at Birmingham, 1300 University Blvd., Birmingham, AL 35233, USA; 5 School of Public Health, University of Alabama at Birmingham, 1665 University Blvd, Birmingham, AL 35294, USA

**Keywords:** caregiver, child, food supply, environment, food and beverages, body weight

## Abstract

**Background/Objectives:** Childhood obesity is prevalent in the United States, and although food environments (FEs) may be related to child weight, findings remain inconsistent. This study evaluated neighborhood and home FEs’ associations with children’s weight and explored interactive effects of the neighborhood and home FEs on child weight. We hypothesized that children exposed to less healthy neighborhood and home FEs would have higher weight. **Methods:** This secondary analysis leveraged baseline data from caregiver–child dyads (*n* = 115) enrolled in an obesity intervention trial (HABITS). The Modified Retail Food Environment Index (mRFEI) was calculated as the proportion of healthy versus unhealthy food vendors within a 4-mile buffer of participants’ homes using food retailer data from business directories and geographic information systems software. Caregivers reported fruit, vegetable, and sugary beverage availability at home. Child weight was measured and used to determine weight-for-age z-scores (WAZs). Linear regression models were used to examine neighborhood FE, home FE, and their interactions as predictors of child WAZs. **Results:** Participants were majority non-Hispanic Black (77%) with household incomes of <$30,000/year (73%). Children were 3.0 ± 1.0 years old. Child WAZs were negatively associated with home fruit availability (β = −0.07, 95% CI: −0.14 to −0.01, *p* = 0.02), with no associations observed for the neighborhood FE or other home FE factors and no interaction between neighborhood and home FEs on weight. **Conclusions:** There were no significant associations between neighborhood FEs and weight among young children, while within the home FE, only the availability of fruit was associated with child weight.

## 1. Introduction

Childhood obesity affects nearly 25% of non-Hispanic Black children in the United States, with low-income populations disproportionately burdened [1,2]. Early-life obesity is associated with a higher likelihood of persistent obesity and increased risk of chronic conditions such as metabolic syndrome, type 2 diabetes, and cardiovascular disease, which also disproportionately affect low-income and Black populations in the Southeastern United States [3,4,5,6,7,8]. Children’s weight status is shaped by a complex interplay of biological, social, structural, and environmental factors and cannot be reduced to individual or household influences alone. Among these broader structural influences, food environments (FEs) reflect structural conditions that determine the availability of foods that may impact children’s dietary patterns and related health trajectories, including weight-related outcomes [9,10]. Evidence suggests that proximal FEs, including neighborhood and home FEs, are associated with children’s diet and eating habits [9,11]. However, existing evidence regarding the associations between these FEs and child weight outcomes remains inconclusive, indicating a need for further research [9]. Importantly, these relationships are complex and should not be interpreted as suggesting that weight status is solely determined by food availability or individual behaviors. However, FEs can shape the dietary opportunities available to children and families and may represent modifiable factors that could support improved dietary patterns and related health outcomes, including weight status, thereby highlighting an important area for intervention and public health research.

While various studies have explored associations between the neighborhood FE and children’s weight, findings are mixed, with some studies showing relationships between neighborhood FE and child weight outcomes and others finding no associations or equivocal ones [12,13,14,15,16]. These inconsistent findings are often attributed to variability in neighborhood FE conceptualization. For instance, past work often defined neighborhood FEs by political or administrative boundaries, like census tracts or counties, despite evidence that such boundaries may not reflect how far people travel for food [17,18,19,20,21]. Additionally, existing studies commonly conceptualize the neighborhood FE by capturing the presence of, or distance to, a single food retailer type (e.g., grocery store or fast food restaurant), thereby narrowly focusing on specific categories of food retailers and failing to account for the overall balance of retailers offering more nutrient-dense options relative to those offering more energy-dense options in the neighborhood FE [9,22,23,24]. Further, much of the existing studies have captured data at a census, county, or state level, thereby limiting the ability to adjust for individual-level confounders, such as household demographics, or more proximal FEs, such as the home FE [25].

The home FE has also been associated with child diet and weight trajectories. In terms of diet, one study found that nearly 90% of food consumed by children 2–5 years old was food available in the home [26]. With regard to weight outcomes, studies have linked greater availability of fruits and vegetables in the home to healthier dietary patterns and, in some cases, differences in child weight status [9]. However, findings on specific household items, such as sugary beverages, are mixed, with most studies reporting no significant association with child weight status [9,27,28]. These inconsistent findings may reflect differences in how the home FE is measured and the populations that have been studied. Studies to date have often relied on food checklists limited to pre-specified items, which may fail to capture the full range of foods available in the home and may provide only limited assessments of the home FE [9,29,30,31]. Additionally, most studies have been conducted outside of the Southeastern United States despite notable disparities in weight-related outcomes in this region [29,30,31,32,33,34].

Overall, the foods and beverages available in the home FE may be related to young children’s weight-related outcomes. However, additional research is needed given inconsistencies reported in the past literature [9,35,36]. Furthermore, one underexplored factor that may help explain the null findings between FEs and children’s weight is the potential interaction between various FEs, namely the neighborhood and home FEs. Additional research that simultaneously evaluates neighborhood and home FEs may help clarify how they are jointly related to child weight [37,38,39].

To address these gaps, this secondary analysis examines the associations between neighborhood and home FEs and relative body weight among young children (ages 1–5 years; predominantly Black) in the Southeast region of the United States. Given the disproportionate burden of obesity among young Black children and health inequities observed in the Southeast region of the United States, examining both neighborhood and home FEs in this population may help identify opportunities to support child health and promote equitable access to health-promoting FEs [1,40]. We hypothesized that food availability across neighborhood and home FEs would be associated with variation in child WAZ. Specifically, we hypothesized that neighborhoods characterized by a greater relative availability of food retailers offering more nutrient-dense options (e.g., grocery stores and supermarkets) compared to retailers offering more energy-dense options (e.g., convenience stores and fast food restaurants) would be associated with lower child WAZs. We further hypothesized that food and beverage availability within the home, including fruits, vegetables, and sugary beverages, would also be associated with variation in child WAZs. We also explored the interaction of the neighborhood FE and each aspect of the home FE on child WAZ.

## 2. Materials and Methods

This secondary analysis leverages baseline (pre-randomization) data from caregiver–child dyads enrolled in the HABITS randomized controlled trial (NCT03433456) [41]. The present analysis of the relationship between FEs and child WAZ was a post hoc exploratory examination and was not part of the parent trial’s pre-specified analytic plan. The parent study, HABITS, was a home-based obesity prevention trial delivered through an existing home visitation program that focused on habit formation and modification of the home environment to support healthy eating and physical activity behaviors among caregiver–child dyads. Participants were randomized to the HABITS intervention or usual care (i.e., continued home visiting without HABITS content). Caregiver and child weight outcomes were compared between the two conditions and served as the primary outcomes of the parent trial. The home FE, anthropometric, and demographic variables included in this secondary analysis were originally collected as part of the HABITS intervention trial. All study procedures were reviewed and approved by the University of Alabama at Birmingham Institutional Review Board, and participants provided written informed consent prior to their inclusion in the study. Additional information regarding study design and procedures has been described in detail in prior publications [41,42].

The parent study, HABITS, enrolled 148 caregiver–child dyads who were receiving services from the First Teacher Home Visiting Program, a federally funded home visiting program serving predominantly low-income families in Alabama [41,43,44]. Participants were recruited from December 2019 through February 2022. However, recruitment and enrollment were halted from March 2020 through September 2020due to COVID-19 restrictions. Caregiver–child dyads were eligible to participate if the child was ≤5 years old and did not have a growth or feeding disorder. Additionally, the caregiver had to be ≥18 years old and could not have lost ≥15 pounds in the past 6 months or be participating in a diet or weight loss program. Families were excluded if they had <1 year of eligibility remaining in the home visiting program or planned to relocate out of the service area within the next year. Interested and eligible caregivers provided informed consent for themselves as well as their child.

Recruitment was as follows: Home visitors introduced the study and provided brief information about it to families during scheduled home visits to assess interest. Home visitors were instructed to approach any of their families that met the following basic criteria: (1) they had completed all home visiting program-specific testing (i.e., testing that is required by the home visiting program to be completed within the first 90 days of a family enrolling in the home visiting program); and (2) the family had at least 1 year of eligibility remaining in the home visiting program. Based on this criteria, home visitors approached a total of 341 families about the study, and of those 134 were not interested in the study. The remaining 207 families were assessed for eligibility. Of those, 59 were excluded for the following reasons: n = 8 did not meeting inclusion criteria, n = 10 declined to participate, *n*-9 were unable to establish contact to complete study enrollment, n = 3 dropped out of the home visiting program after screening/before they could be enrolled, and n = 29 became ineligible/dropped out of home visiting program during COVID-19 shutdown/before they could be enrolled. The remaining 148 families were consented/completed baseline assessment for the study.

While home FE, anthropometric, and demographic measures included in these secondary analyses were collected as part of the original trial, measures of the neighborhood FE were collected following the completion of the HABITS trial to characterize each family’s neighborhood FE. Food retailers around participants’ homes were identified using the business directory (Simply Analytics Inc., New York, NY, USA; https://simplyanalytics.com/, accessed on 1 September 2025). Simply Analytics sources their data from official government agencies (e.g., the United States Census Bureau) and private business directories (e.g., Dun and Bradstreet). We conducted searches to identify the food retailer types, which are reported in Table A1. The following information on food retailers was obtained: food retailer name, address, number of employees, the year the retailer opened, and North American Industry Classification System code (NAICS) [45]. NAICS codes are six-digit codes used to determine store types (grocery store, fast food restaurant, etc.). After compiling the list of relevant food retailers, data were analyzed to identify and exclude stores that (1) did not have a primary NAICS code that aligned with the codes provided in Table A1, (2) were duplicates, or (3) opened after the completion of the HABITS baseline data collection.

Participant addresses and food retailer locations were imported to ArcGIS Pro, a geographic information system application that allows visualization and spatial analysis of FEs. Based on findings from the United States Department of Agriculture’s (USDA) national Food Acquisition and Purchasing Survey, which indicated that most individuals obtain most of their food within four miles of their home, the neighborhood FE was defined as a four-mile straight-line buffer around each participant’s home [19]. Within each buffer we obtained counts of each type of food retailer (Table A1) with the “Spatial Join Analysis” tool in ArcGIS Pro version 3.4.

To characterize the relative healthiness of families’ neighborhood FEs, we used the Modified Retail FE Index (mRFEI) created by the Centers for Disease Control and Prevention (CDC) [46]. The mRFEI is a ratio of healthy to all food retailers in an area, with a higher score indicating a healthier FE. For the mRFEI, healthy food retailers are defined as warehouse clubs and supercenters, supermarkets, larger grocery stores, and fruit and vegetable retailers. Unhealthy food retailers are defined as convenience stores, limited-service restaurants, and small grocery stores (grocers with ≤3 employees). Each food retailer is specifically classified by their NAICS code. Table A1 further shows the mRFEI store classification. The formula to calculate the neighborhood mRFEI is as follows: (the number of healthy food retailers, divided by total retailers) multiplied by 100. mRFEI scores range from 0 to 100, with 100 being the healthiest possible neighborhood FE. As an additional means of describing the FE, we used Food Access Research Atlas data to determine if the participants resided in rural versus urban areas and if they lived in food deserts [47]. As determined by the USDA, individuals were considered as living in a food desert if they meet the criteria for both a low-income area and low-access area [47].

The home FE was captured with a HABITS-modified Home Food and Activity Environment (HFA) assessment, which has been previously described [41,42]. Briefly, the HFA assessment was administered by telephone. Trained research staff asked caregivers to report all fruits, vegetables, and sugary beverages present in their home. This included all fresh, frozen, and canned fruits and vegetables as well as the following categories of sugary beverages in the home: soda, energy/sports drinks, sweet teas, coffee drinks, flavored milks, drink powders or mixes, juice, and frozen juice cans. The HFA assessed the variety of foods available as opposed to the quantity of foods. For example, if a household had a bag of apples, a carton of fresh raspberries, and 2 bags of frozen raspberries, this was counted as two fresh fruits and one frozen fruit in the home, with the cumulative total fruit score being 3 fruits.

Study staff measured child and caregiver weight in duplicate with a Tanita scale (Model BWB-800S, Tanita, Portage, ML, USA) to the nearest 0.1 kg. For younger children who could not stand on the scale alone, the caregivers held their child during the weight measure, and the caregiver’s weight was then subtracted to obtain the child’s weight. Because the study was conducted in the midst of the COVID-19 pandemic and study staff were practicing social distancing recommendations, height/length measurements were not obtained for children and caregiver height was self-reported. Caregiver BMI was calculated. Child weight status was assessed using WAZ, as length/height measurements were not available due to COVID-19-related data collection constraints. While the WAZ is not a substitute for BMI and does not directly measure adiposity, prior work demonstrates that the WAZ can serve as an initial weight status screening when height is unavailable [48]. Child WAZs were determined using growth reference charts [49,50]. As recommended, World Health Organization (WHO) growth charts were used for children ≤ 2 years old and Centers for Disease Control and Prevention (CDC) growth charts for children > 2 years old [51,52].

Questionnaires were used to capture demographics for the household, such as number of adults and children in the home, annual household income, days since someone in the home last went grocery shopping, and use of the Supplemental Nutrition Assistance Program (SNAP) and the Special Supplemental Nutrition Program for Women, Infants, and Children (WIC). Questionnaires also gathered information about the caregiver, including caregiver age, race/ethnicity, education, and marital status. Caregivers also reported information regarding the child, including child birthweight, the age the child was introduced to complementary foods, child race/ethnicity, child age, breastfeeding history, and child physical activity level.

Descriptive statistics (mean, standard deviation, and frequency) were calculated for caregiver and child demographic characteristics and anthropometrics and to characterize the neighborhood FE and the food items available in the home. Missing data were examined for all variables by calculating the frequency and proportion of missing values. To assess potential differences, characteristics of participants included in the final analytic sample were compared with those excluded due to missing data using independent *t*-tests for continuous variables and chi-square tests for categorical variables. Linear regression models were performed to evaluate the relationships between the neighborhood FE (mRFEI) and child WAZ. Secondary models were also conducted using healthy and unhealthy food retailer measures as separate predictor variables to explore their individual associations with child WAZ. Three separate linear regression models were conducted to assess associations between the following aspects of the home FE and WAZ: (1) fruit availability, (2) vegetable availability, and (3) sugary beverage availability. We also conducted exploratory analyses to assess the interactive effects of the home and neighborhood FEs on child WAZ. Moderation analyses were conducted using Andrew Hayes’ PROCESS macro (Andrew F. Hayes; https://www.processmacro.org/, accessed 1 September 2025), a tool for testing mediation and moderation relationships using regression-based methods [53]. Additionally, given developmental differences in the ability to self-feed and obtain food outside the home between children aged 1–3 years and those aged 3–5 years, we conducted sensitivity analyses in which regression models were stratified by age group (1–3 years vs. 3–5 years). We also conducted descriptive analyses stratified by SNAP participation to examine differences in FE variables and child WAZ among SNAP and non-SNAP households using independent *t*-tests.

To achieve the most parsimonious models, exploratory analyses of potential covariates were conducted for each linear regression model. Covariate selection for the final multivariable linear regression models was conducted using a theory-driven and statistically guided approach. Candidate covariates were identified *a priori* based on the existing literature, including variables previously shown to be associated with child weight outcomes and/or the neighborhood or home FEs [54]. Potential covariates were as follows: child birthweight (kg) [55], child breastfeeding history [56] (was breastfed/was never breastfed), age of introduction to complementary foods [57], child physical activity level [58] (dichotomized as engaging in physical activity fewer than 4 times per day versus 4 or more times per day), child race [59], child age [60], caregiver age [61], caregiver BMI [57], marital status [62] (single/married or living with significant other), education attainment [57] (≤high school graduate or >high school graduate), household composition [62,63,64,65] (number of adults and children in the home), household income [66,67] (<$30,000/≥$30,000), days since the family last went grocery shopping, SNAP benefit participation status (participated/did not participate) [68], household urbanicity/rurality [69], and food desert status [70]. To supplement the theoretical framework, bivariate analyses were performed to assess the unadjusted associations between each potential covariate and the primary outcome. Variables were evaluated using correlation matrices. Covariates demonstrating a statistically significant association with the outcome at a threshold of *p* < 0.10 were included in the final models. The primary predictor of each model remained in the models regardless of correlation with outcome [54]. All categorical variables were dummy coded for the regression model. Testing was performed to verify that all assumptions of multiple linear regression were met. Statistical analyses were performed utilizing SAS software (version 9.4, 2002e2012 by SAS Institute Inc., Cary, NC, USA) with significance set as *p* < 0.05.

## 3. Results

### 3.1. Participant Demographics and Food Environment Characteristics

The parent study, HABITS, recruited a total sample of 148 caregiver–child dyads with children ranging in age from 5 weeks to 5 years old. For this secondary analysis, we excluded children < 1 year old (n = 17) because their diet primarily consists of breastmilk/formula rather than the foods present in neighborhood or home FEs. Of the 131 children 1 year of age or older, n = 16 were excluded for missing covariate data, including: child birthweight (n = 7), breastfeeding history (n = 8), and caregiver BMI (n = 1). The final analytic sample consisted of n = 115 caregiver–child dyads, which is the sample reflected in all subsequent text and tables. Forty-nine percent of the children were female and 77% were non-Hispanic Black. Additional demographics for family and household demographics can be found in Table 1. There were no statistically significant differences between participants with missing data and those included in the analytic sample for any characteristics reported in Table 1.

**Figure 1:**
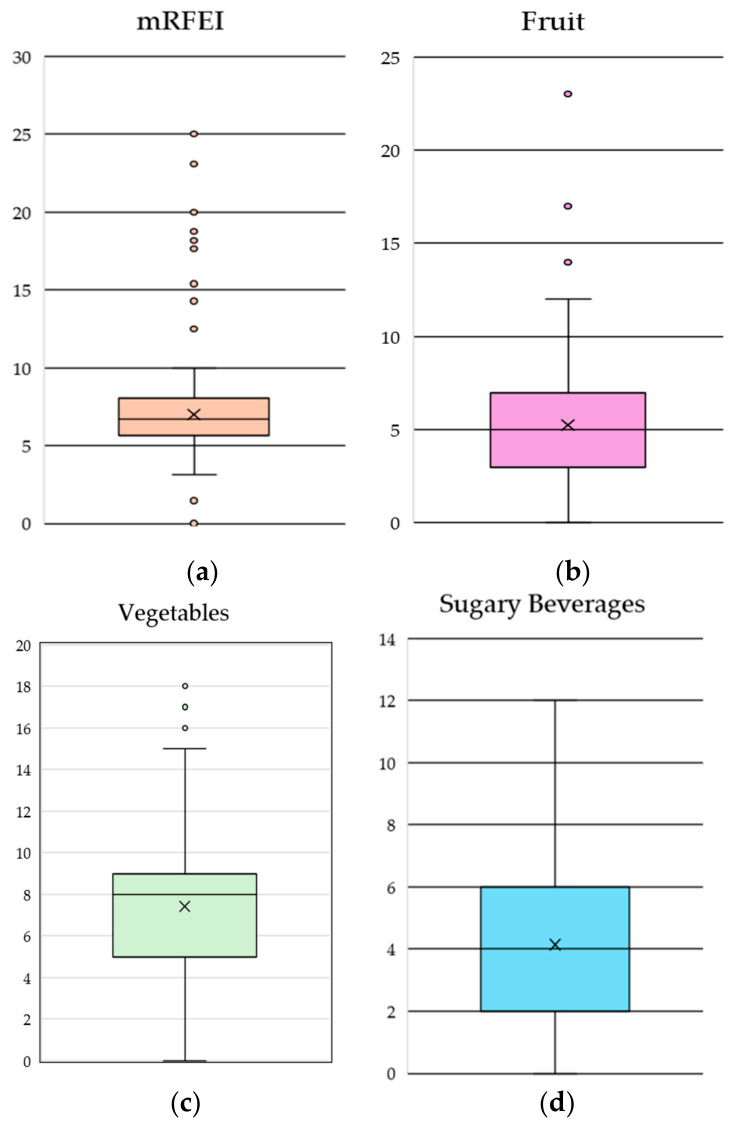
Distribution of food environment measures. (**a**) Modified Retail Food Environment Index (mRFEI). (**b**) Home fruit availability. (**c**) Home vegetable availability. (**d**) Home sugary beverage availability. Colored boxes are used to distinguish the four measures (**a**–**d**). The box represents the interquartile range, the horizontal line represents the median, the “x” denotes the mean, whiskers represent the range of non-outlier values, and circles indicate outliers.

The distribution of FE variables among the samples are shown in Figure 1. Participants had between 0 and 23 fruits, 0 and 18 vegetables and 0 and 12 sugary beverages. In the neighborhood FE, the number of healthy food retailers ranged from 0–23, and unhealthy retailers ranged from 0–296. Considering the healthiness of neighborhood FEs, families’ mRFEI score ranged from 0–25, with a score of 100 indicating the healthiest neighborhood FE. Additional information regarding FE can be found in Table 1.

### 3.2. Associations Between Child Weight and Food Environments

Associations between FEs and child weight are reported in Table 2. When adjusting for child birthweight, breastfeeding history, caregiver race, and caregiver BMI, the neighborhood FE was not associated with child WAZ (β = 0.0008, CI: −0.04 to 0.04, *p* = 0.97; Table 2, **Model 1**). Results were unchanged in age-stratified analyses. When separate models were run stratified by age, the inverse association between fruit availability and WAZ remained statistically significant among children aged 1 to <3 years (95% CI: −0.06 to 0.05; *p* = 0.87; Table A2), whereas among children aged 3–5 years, the estimated association was 0.004 WAZ units (95% CI: −0.07 to 0.07; *p* = 0.92; Table A2). When models were run separately for healthy food retailer count or unhealthy food retailer count as the neighborhood FE predictor of interest, neither heathy (β = 0.01, 95% CI: −0.02 to 0.04, *p* = 0.46; Table A3) nor unhealthy (β = 0.001 95% CI: -0.001 to 0.003, *p* = 0.36; Table A3) food retailers were significantly associated with child WAZ.

Only fruit availability in the home was associated with child WAZ (β = −0.07, 95% CI: −0.14 to −0.01, *p* = 0.02; Table 2, Model 2) when controlling birthweight, breastfeeding history, caregiver race, and caregiver BMI. Specifically, living in a home with a greater availability of fruits was associated with lower child WAZ. When separate models were run stratified by age, the inverse association between fruit availability and WAZ remained statistically significant among children aged 1 to <3 years (β = −0.09, 95% CI: −0.17 to −0.01, *p* = 0.03; Table A2) but was not observed among children aged ≥3 years (β = −0.08, 95% CI: −0.19 to 0.02, *p* = 0.11; Table A2). In fully adjusted models, neither vegetable availability (β = −0.03, 95% CI: −0.10 to 0.03, *p* = 0.31; Table 2, Model 3) nor sugary beverage availability (β = −0.02, 95% CI: −0.11 to 0.06, *p* = 0.60; Table 2, Model 4) were significantly associated with child WAZ. The associations between vegetable and sugary beverage availability and child WAZ remained non-significant after stratifying by age (Table A2). Sensitivity analyses were conducted for each model (Model 1–4; Table 2) to determine whether the associations persisted after excluding pregnant caregivers (n = 15). Results were unchanged, so pregnant caregivers were retained in the final models reported in Table 2. Results remain unchanged even after adjusting for other relevant covariates including days since last grocery shopping, age at introduction of complementary foods, and child physical activity. Furthermore, after adjusting for birthweight, breastfeeding history, caregiver race, and caregiver BMI, no significant interactions were observed between neighborhood FE and fruit (β = 0.0006, 95% CI: −0.01 to 0.01, *p* = 0.93), vegetable (β = −0.01, 95% CI: −0.02 to 0.003, *p* = 0.14), or sugary beverage availability (β = 0.002, 95% CI: −0.02 to 0.02, *p* = 0.86) in relation to child WAZ.

### 3.3. Exploring Associations Between SNAP Participation and Food Environments

When comparing the FEs of SNAP and non-SNAP recipients, there were no statistically significant differences in the neighborhood FE. Within the home, the non-SNAP user group had greater home fruit availability (7.41 ± 3.81 vs. 4.53 ± 3.13, *p* < 0.0001; Table A4), but there were no differences in vegetable and sugary beverage availability. In exploratory analyses including SNAP participation in the linear regression models, adjustment for SNAP group did not change the results for associations between the FE and child WAZ.

## 4. Discussion

This study evaluated the associations between neighborhood FE (mRFEI), home food availability (fruits, vegetables, and sugary beverages), and young children’s WAZ. Consistent with our hypothesis, we found that increased fruit availability in the home was associated with a lower child WAZ. However, there were no significant associations between the other elements of the home FE (vegetable and sugary beverage availability in the home) and child WAZ. Additionally, there was no significant association between neighborhood FE and children’s WAZ nor a significant interaction between neighborhood and home FEs on child WAZ.

We found that fruit availability in the home was negatively associated with child WAZ, such that each additional fruit available in the home was associated with a 0.07 lower WAZ. While we did not assess changes in children’s weight over time, these findings contribute to a growing body of literature examining how home FEs relate to child dietary patterns and weight-related outcomes. Prior research suggests that even modest differences in BMI z-scores (0.10) have been associated with improvements in metabolic health outcomes [71,72]. Our findings suggest that greater fruit availability in the home may support dietary patterns among young children that are associated with improved diet quality, with potential implications on weight-related outcomes.

In age-stratified analyses, the inverse association between fruit availability and WAZ remained statistically significant among children aged 1–<3 years old but was not observed among children aged ≥3 years old. These findings suggest that the observed association may be primarily driven by younger children, for whom household food availability more directly shapes dietary intake, whereas the lack of association among older children may reflect growing dietary autonomy and external influences. However, these results should be interpreted with caution, as the magnitude of the association was similar across age-stratified models, and we may have been underpowered to detect an association among the subset of children ≥ 3 years of age. Overall, prior research has linked higher fruit availability and intake with improved diet quality in children, which may contribute to healthier weight status, as fruits are typically lower in energy density and may displace more energy-dense foods [9,25]. However, given the cross-sectional nature of this study, these findings should be interpreted cautiously. Additionally, weight-related measures represent only one dimension of health and should be interpreted alongside broader indicators of dietary and health outcomes.

Vegetable availability was not significantly associated with child weight in our study population. Vegetables are generally less accepted among children when compared to fruits, and increasing their consumption often requires repeated exposure [73,74]. As such, even when vegetables are present in the home, it is possible that children’s vegetable intake may not be sufficient to detect associations with weight-related outcomes. For our population, disparities in vegetable intake among Black children likely reflect broader structural and environmental inequities that influence food availability, preferences, and exposure [75]. Future research is needed to understand how vegetables in the home may relate to actual dietary intake among young children from diverse backgrounds and, in turn, weight outcomes.

Contrary to past work, we did not detect significant associations between sugary beverage availability in the home and child WAZ. These findings could be due to the methodology used to capture sugary beverages in the home [9,76]. Our assessment of food availability captured the variety rather than quantity of items available. For instance, a family could have had several cases of the same soda in the home; however, this would only count as one type of sugary beverage in the home. Given the evidence linking sugary beverage consumption to child weight, measuring sugary beverage quantities in the home, rather than availability, may better explain the sugary beverage association with child weight [76].

Informed by gaps in prior research, this study accounted for the relative healthiness of the neighborhood FE in addition to focusing on individual types of food retailers in isolation [9,77]. We also defined the boundary of the neighborhood FE based on past research that evaluated how far people traveled for food rather than politically defined boundaries (census tract/block, counties, city lines), which may not be representative of where individuals obtain food [20,23,78]. We did not detect associations between the neighborhood FE and child weight in this population. Considering that most of the study population utilized SNAP benefits, the lack of observed association in this study may be attributable to insufficient information on whether retailers within the neighborhood FE were authorized SNAP vendors, particularly in light of prior research demonstrating associations between access to SNAP-authorized retailers and diet quality among SNAP participants [79]. The association of SNAP with diet quality is consistent with our finding that households receiving SNAP benefits had significantly higher fruit availability compared to non-SNAP households. This suggests that SNAP participation may have helped support improved access to fruits, contributing to higher fruit availability among these households. It is important to note that the present study was carried out during the COVID-19 pandemic when SNAP allotments were temporarily increased, which could have impacted these findings. Nonetheless, it will be important for future research examining how FEs impact child weight outcomes to consider the role of SNAP participation and the presence of SNAP-authorized retailers within neighborhood environments on outcomes.

Our null findings for the relationship between the neighborhood FE and child weight may be explained by the young age of the children in this sample, as younger children may lack the autonomy needed to be influenced by neighborhood FEs. Past work looking at the influence of the neighborhood FE on children’s weight has included children across a large age range (birth–19 years old), where older children have greater independence in food acquisition [9,12,39]. In contrast, children aged 1–5 years remain highly dependent on caregivers for food provision, which may explain why the home FE appears more relevant to weight-related outcomes in this population. These findings underscore the importance of considering developmental stage when evaluating the influence of FEs on early childhood weight.

Additionally, the lack of an observed association may reflect the limited variability in neighborhood FE healthfulness, which could have reduced our ability to detect an effect. While the mRFEI index ranges from 0 (least healthy) to 100 (healthiest), our sample’s mRFEI values ranged from 0 to 25 [46]. Notably, the low mRFEI scores observed in the present study were largely driven by an abundance of unhealthy food retailers rather than a lack of available healthy food retailers around participants’ homes, which is consistent with prior studies characterizing FEs of predominantly Black neighborhoods [80]. Considering how our samples’ low mRFEI scores were largely driven by an overabundance of unhealthy retailers rather than limited access to healthy retailers, our findings support past research advocating for a need to account for both healthy and less healthy food stores to understand the neighborhood FE [81]. For future investigation, the mRFEI remains a useful tool, as it accounts for overall healthiness but also offers the opportunity to explore healthy and unhealthy food retailers independently.

Alternatively, as the study population was predominantly low income, the presence of retailers in their neighborhood FE may not have reflected the families’ interactions with these retailers [82,83]. For instance, children could have been residing in neighborhoods with healthy food retailers, but economic constraints may have limited families’ ability to purchase food from those retailers, resulting in null associations between retailer type and child weight. Another possibility is that, because our sample included families receiving government assistance for food purchases, many households may have been food secure, which could have limited our ability to detect an association with child weight outcomes. Indeed, results from a previous study found that residing in a less healthy neighborhood FE was significantly and positively associated with child weight only among households with food insecurity, but there was no association between neighborhood FE and child weight outcomes among food-secure households [84]. While not measured in the present study, future studies should consider how household food security could modify associations between the neighborhood FE and child weight. Taken together, these findings should not be interpreted as evidence that neighborhood FEs are unimportant but rather that associations may depend on how environments are measured, the outcomes assessed, and the characteristics of the study population.

Our study addresses several gaps by examining both neighborhood and home FEs, leveraging individual-level data, and adjusting for key confounders like socioeconomic status, birth weight, breastfeeding history, and caregiver BMI, variables that have been strongly related to child weight but are often unavailable in neighborhood-level studies. Additionally, our sample included a high proportion of non-Hispanic Black children from low-income families in Alabama, a population that warrants further investigation given the limited evidence on the relationship between their FEs and weight, as well as the recognized public health concern of obesity among this population. While this study offers insights into how FEs may be related to child weight, there are several limitations that should be addressed. First, this cross-sectional analysis captures the FE, child weight, and covariates at a single point in time, which limits the ability to establish directionality of observed associations. This limitation introduces the potential for reverse causation and restricts a causal interpretation of the observed associations, including the relationship between fruit availability and child WAZ.

Furthermore, assessments of the home FE and demographic characteristics were not conducted using validated instruments, but rather, individual items were adapted from validated measures to align with the goals of the parent study [33]. Notably, results remained unchanged even when the HABITS study home FE inventory was scored using a preexisting validated measure. Additionally, our measures of FEs were relatively crude and captured the availability rather than utilization of FEs. For example, the neighborhood FE food retailer types (e.g., grocery stores, fast-food restaurants, etc.) were used as proxies for the availability of healthy or unhealthy foods, which may not fully capture the availability, affordability, or quality of foods offered within those retailers. Further, home food availability measures did not capture quantities of foods or actual consumption patterns. It is also a limitation that home FE measures were self-reported rather than objectively assessed by study staff, which may have introduced self-report bias, including potential social desirability and recall bias. These measurement limitations may have attenuated associations and reduced our ability to detect meaningful relationships between FEs and weight outcomes among the children. Furthermore, because most participants utilized SNAP benefits, a study limitation is that we lacked information on whether food retailers in their neighborhoods were SNAP-authorized. Additionally, there are known limitations to using business directories to obtain counts of food retailers, including accuracy of food retailer identification and classification. Another limitation of the study was that it was conducted during the midst of the COVID-19 pandemic. Both physical and monetary access to foods (stores closed, lost income, changes in food assistance, etc.) may have impacted the participants’ FEs.

Although models were adjusted for key covariates, residual confounding remains possible due to unmeasured factors such as caregiver feeding practices, household food preferences, and cultural influences, which may have influenced both food availability and child weight outcomes. Additionally, due to social distancing protocols during the pandemic, HABITS study staff were unable to obtain child length/height measurements and, in some cases, could not measure caregiver height, resulting in reliance on self-reported caregiver height. Consequently, analyses were limited to child WAZ, preventing assessment of overall child size. While the WAZ is informative for examining patterns of relative weight status within the study sample, particularly when other anthropometric data are unavailable, it does not account for height. Accordingly, these findings should be interpreted with caution, as the WAZ does not directly assess adiposity. Future studies incorporating child BMI z-scores or other validated measures of adiposity are needed to further examine these associations.

In terms of the sample, selection bias is also a potential limitation. Because all participants were enrolled in a home visiting program, the sample may represent families who are more engaged with services than the broader population. As a result, findings may not be generalizable to families outside of the home visiting context, and observed associations between FEs and child weight may differ in other populations. Additionally, our findings should be interpreted within the context of the study sample, which primarily consisted of low-income, non-Hispanic Black children residing in the Southeast region of the United States. As such, the results may have limited generalizability to populations with different racial, socioeconomic, or geographic characteristics. Considering the statistical analyses, the study may have been underpowered to detect interaction effects between neighborhood and home food environments on child WAZ because of the relatively small sample size and limited variability in neighborhood food environment measures. Additionally, because multiple related statistical tests were conducted, there is an increased risk of Type I error. Consistent with recommendations from the literature, we sought to mitigate this concern by emphasizing effect sizes rather than relying solely on statistical significance, interpreting findings cautiously, and framing the observed associations as preliminary rather than definitive. Furthermore, we used a composite measure of the neighborhood FE to reduce the number of comparisons where possible. However, a comparable composite measure was not available for the home FE variables. Therefore, these findings should be interpreted with caution and warrant confirmation in larger studies.

## 5. Conclusions

In this study of a predominantly low-income Black population in the Southeastern region of the United States, neighborhood FEs were not associated with child weight, while within the home environment, fruit availability was the only factor significantly associated with child weight. Specifically, greater availability of fruit in the home was associated with lower WAZ. When considered alongside prior literature, our findings suggest that addressing modifiable characteristics of the home FE, particularly fruit availability, may represent a practical target for interventions aimed at supporting patterns of relative body weight and diet quality in early childhood within our population. The null findings for other home food indicators, including vegetables and sugary beverages, should be interpreted with caution, as they may reflect limitations in measurement rather than a true absence of association. Given the mixed findings regarding FEs and children’s weight observed in this study and prior research, further investigation is warranted to better understand the relationship between neighborhood and home FEs and children’s weight, particularly across diverse populations. Future studies should include a broader evaluation of food groups available in the home (e.g., grains, meats, dairy, ultra-processed foods) and utilize more rigorous assessment methods, such as measuring quantities of foods and conducting in-home evaluations by trained research staff. Additionally, future work would benefit from examining interactions within neighborhood and home FEs rather than focusing solely on food/food retailer availability.

## Figures and Tables

**Table 1:** Participant characteristics by missing-data status.

	Total Sample (n = 131)	Final Sample (n = 115)	Excluded Sample (n = 16)
Caregiver age (years)	32.7 ± 8.3	32.2 ± 7.0	36.3 ± 14.5
Child age (years)	2.9 ± 1.00	3.0 ± 1.0	2.7± 1.2
**Caregiver race**			
Black, non-Hispanic	100 (76)	88 (77)	12 (75)
White, non-Hispanic	31 (24)	27 (23)	4 (25)
**Caregiver education**			
High school graduate	81 (62)	43 (37)	9 (56)
Some college or above	50 (38)	72 (63)	7 (44)
**Marital status**			
Married or living with significant other	80(61)	73 (61)	9 (56)
Single	51 (39)	42 (39)	7 (44)
**Household income ^a^**			
<$30,000	90 (72)	80 (73)	10 (67)
≥$30,000	35 (28)	30 (27)	5 (33)
Days since last went grocery shopping	8.2 ± 11.9	8.0 ± 12.0	10.3 ± 5.7
Caregiver Body Mass Index (kg/m^2^)	34.2 ± 10.0	34.0 ± 10.1	38.6 ± 9.0
Child weight-for-age z-Score	0.4 ± 1.3	0.3 ± 1.2	0.7 ± 1.6
Child birthweight (kg)	3.1 ± 0.6	3.1 ± 0.6	
Child age of introduction to complementary foods (months)	5.2 ± 3.5	6.7 ± 2.9	5.4 ± 2.4
Child physical activity ^b^	69 (53)	60 (52)	7 (44)
Number of adults in the home	1.8 ± 0.8	2.5 ± 1.3	1.7 ± 0.7
Number of children in the home	2.4 ± 1.3	1.8 ± 0.9	2.4 ± 1.5
Used SNAP benefits ^c^	95 (74)	85 (75)	10 (63)
Used WIC benefits ^d^	88 (68)	75 (66)	13 (81)
Lived in an urban area ^e^	99 (76)	85 (75)	13 (81)
Lived in a food desert ^f^	53 (41)	43 (38)	10 (63)
Modified Retail FE Index ^g^	7.0 ± 4.8	7.1 ± 5.1	6.4 ± 2.7
Healthy retailers ^h^	10.6 ± 7.8	10.4 ± 7.9	12.0 ± 6.4
Unhealthy retailers ^i^	142.9 ± 101.9	141.5 ± 105.2	152.5 ± 76
Fruit availability in the home	5.2 ± 3.5	5.3 ± 3.6	5.0 ± 3.3
Vegetable availability in the home	7.4 ± 3.7	7.6 ± 3.6	5.8 ± 3.9
Sugary beverage availability in the home	4.1 ± 2.6	4.3 ± 2.6	3.1 ± 2.5

Exclusions were based on missing data for variables included in the final linear regression models. ^a^ n = 5 missing data for income variable (not reported). ^b^ Child physical activity dichotomized: 0 = engaging in physical activity fewer than 4 times per day versus 1 = engaging in physical activity more times per day. ^c^ n = 2 missing data for SNAP benefit usage (not reported). ^d^ n = 2 missing data for WIC benefit usage (not reported). ^e^ Urbanicity dichotomized: 0 = participant lived in a rural census tract; 1 = participant lived in an urban census tract (n = 1 missing data point for urban status). ^f^ Food desert status dichotomized: 0 = participant did not live in a food desert; 1 = participant lived in a food desert (n = 1 missing data point for food desert status). ^g^ Relative healthiness of neighborhood FE (the number of healthy food retailers, divided by total retailers) multiplied by 100. ^h^ Healthy food retailers included supermarkets, large grocery stores, warehouse clubs, supercenters, and fruit and vegetable retailers (stores further defined in Table A1). ^i^ Unhealthy food retailers included convenience stores, gas stations, limited-service restaurants, and small grocery stores (stores further defined in Table A1).

**Table 2:** Adjusted multiple linear regression analysis evaluating the relationship between child weight-for-age z-scores and the neighborhood and home food environments.

			95% CI	
	β Coefficients	*p* Value	Lower	Upper
**Neighborhood Food Environment**				
**Model 1**: **(Y) child weight-for-age z-score (n = 115)**	Adjusted R^2^ = 0.12			
Child birthweight (kg)	0.42	0.02 *	0.07	0.78
Child breastfeeding history **^a^**	−0.48	0.04 *	−0.92	−0.03
Caregiver race **^b^**	−0.12	0.67	−0.65	0.42
Caregiver BMI	0.03	0.005 *	0.01	0.05
Neighborhood food retailer availability (mRFEI)	0.0008	0.97	−0.04	0.04
Home Food Environment				
**Model 2**: **(Y) child weight-for-age z-score (n = 115)**	Adjusted R2 = 0.16			
Child birthweight (kg)	0.47	0.009 *	0.12	0.82
Child breastfeeding history **^a^**	−0.44	0.05 *	−0.87	−0.004
Caregiver race **^b^**	−0.30	0.27	−0.84	0.23
Caregiver BMI	0.03	0.003 *	0.01	0.05
Home fruit availability	−0.07	0.02 *	−0.14	−0.01
**Model 3**: **(Y) child weight-for-age z-score (n = 115)**	Adjusted R2 = 0.13			
Child birthweight (kg)	0.47	0.01 *	0.10	0.83
Child breastfeeding history **^a^**	−0.43	0.06	−0.88	0.03
Caregiver race **^b^**	−0.16	0.54	−0.70	0.37
Caregiver BMI	0.03	0.005 *	0.01	0.05
Home vegetable availability	−0.03	0.31	−0.10	0.03
**Model 4: (Y) child weight-for-age z-score (n = 115)**	Adjusted R2 = 0.12			
Child birthweight (kg)	0.43	0.02 *	0.07	0.78
Child breastfeeding history **^a^**	−0.47	0.04 *	−0.92	−0.03
Caregiver race **^b^**	−0.09	0.73	−0.62	0.44
Caregiver BMI	0.03	0.007 *	0.009	0.05
Home sugary beverage availability	−0.02	0.60	−0.11	0.06

All β coefficients are unstandardized. ^a^ Child breastfeeding history dichotomized as 0 = child was never breastfed and 1 = child was breastfed at least once. ^b^ Caregiver race dichotomized (0 = caregiver identified as anything besides non-Hispanic Black; 1 = caregiver is non-Hispanic Black). * Indicates statistical significance at *p* < 0.05.

## Data Availability

The data presented in this study are available on request from the corresponding author. Data are not publicly available due to privacy restrictions.

## Abbreviations

The following abbreviations are used in this manuscript:

BMI	Body Mass Index
CDC	Centers for Disease Control and Prevention
FE	Food Environment
HFA	Home Food Assessment
mRFEI	Modified Retail Food Environment Index
NAICS	North American Industry Classification System
SNAP	Supplemental Nutrition Assistance Program
USDA	United States Department of Agriculture
WAZ	Weight-for-age z-score
WIC	Special Supplemental Nutrition Program for Women, Infants, and Children
WHO	World Health Organization

## Appendix A

**Table A1:** Food retailer classification and Modified Retail Food Environment Index (mRFEI) formula.

	Food Outlet Type	NAICS *	Additional Descriptives
Healthy Food Retailers	Supermarkets	445110	≥50 annual payroll employees.
	Larger grocery stores	445110	Food stores with 10–49 payroll employees.
	Warehouse clubs and supercenters	455211	Warehouse clubs and supercenters.
	Fruit and vegetable retailers	445230	Including permanent produce stands.
Unhealthy Food Retailers	Convenience stores: gas stations	457110	Convenience food with gasoline stations. Gasoline stations with convenience stores.
	Convenience stores: All other general merchandise retailers	455219	These establishments retail a general line of new and used merchandise, such as apparel, automotive parts, dry goods, groceries, hardware, houseware, or home furnishings, and other lines in limited amounts, with none of the lines predominating.
	Limited-service restaurants	722513	Establishments where customers typically order and pay before being served. Food may be consumed on-premises, taken out, or delivered.
	Small grocery stores	445110	≤3 annual payroll employees.

* **(NAICS)** North American Industry Classification Codes classify businesses by 6-digit codes. These codes determine store types (grocery store, fast food restaurant, etc.).

**Table A2:** Multiple linear regression of food environment factors and child weight status, stratified by age (1–<3 vs. 3–5 years).

		Age 1–<3 Years (n = 63)				Age 3–5 Years (n = 52)			
				95% CI				95% CI	
Model		β Coefficients	*p* Value	Lower	Upper	β Coefficients	*p* Value	Lower	Upper
**Neighborhood Food Environment**									
**1**	**(Y) Child Weight for age** **z-score**	Adjusted R^2^ = 0.10				Adjusted R^2^ = 0.15			
	Child birthweight (kg)	0.39	0.09	−0.07	0.86	0.43	0.14	−0.15	1.02
	Child breastfeeding history **^a^**	−0.70	0.03 *	−1.34	−0.06	−0.27	0.42	−0.93	0.40
	Caregiver race **^b^**	0.37	0.14	−1.31	0.18	0.46	0.23	−0.30	1.23
	Caregiver BMI	0.03	0.12	−0.007	0.06	0.04	0.02 *	0.007	0.07
	Neighborhood food retailer availability (mRFEI)	−0.005	0.87	−0.06	0.05	0.004	0.92	−0.07	0.07
**Home Food Environment**									
**2**	**(Y) Child weight-for-age** **z-score**	Adjusted R^2^ = 0.18				Adjusted R^2^ = 0.20			
	Child birthweight (kg)	0.45	0.05 *	0.009	0.90	0.45	0.12	−0.12	1.01
	Child breastfeeding history **^a^**	−0.70	0.03 *	−1.31	−0.09	−0.18	0.58	−0.81	0.46
	Caregiver race **^b^**	−0.91	0.02 *	−1.68	−0.14	0.38	0.31	−0.37	1.12
	Caregiver BMI	0.03	0.06	−0.0009	0.06	0.04	0.01 *	0.008	0.06
	Home fruit availability	−0.09	0.03 *	−0.17	−0.01	−0.08	0.11	−0.19	0.02
**3**	**(Y) Child weight-for-age** **z-score**	Adjusted R^2^ = 0.10				Adjusted R^2^ = 0.19			
	Child birthweight (kg)	0.40	0.10	−0.07	0.87	0.53	0.07	−0.05 *	1.12
	Child breastfeeding history **^a^**	−0.69	0.04 *	−1.34	−0.03	−0.17	0.60	−0.81	0.48
	Caregiver race **^b^**	−0.57	0.14	−1.33	0.19	0.39	0.30	−0.36	1.13
	Caregiver BMI	0.028	0.11	−0.006	0.06	0.04	0.01	0.008	0.06
	Home vegetable availability	−0.008	0.85	−0.10	0.08	−0.07	0.12	−0.17	0.02
**4**	**(Y) Child weight-for-age** **z-score**	Adjusted R^2^ = 0.11				Adjusted R^2^ = 0.20			
	Child birthweight (kg)	0.37	0.12	−0.10	0.83	0.37	0.20	−0.20	0.94
	Child breastfeeding history **^a^**	−0.68	0.04 *	−1.32	−0.04	−0.16	0.61	−0.80	0.48
	Caregiver race **^b^**	−0.56	0.13	−1.30	0.18	0.65	0.09	−0.16	1.42
	Caregiver BMI	0.03	0.10	−0.005	0.06	0.03 *	0.03	0.004	0.06
	Home sugary beverage availability	0.04	0.56	−0.09	0.17	−0.09	0.09	−0.20	0.02

All β coefficients are unstandardized. ^a^ Child breastfeeding history dichotomized as 0 = child was never breastfed and 1 = child was breastfed at least once. ^b^ Caregiver race dichotomized (0 = caregiver identified as anything besides non-Hispanic Black and 1 = caregiver is non-Hispanic Black). * Indicates statistical significance at *p* < 0.05.

**Table A3:** Adjusted multiple linear regression examining the association between healthy and unhealthy food retailers and child weight-for-age z-scores.

				95% CI	
Model		β Coefficients	*p* Value	Lower	Upper
**1**	**(Y) Child weight-for-age** **z-score (n = 115)**	Adjusted R^2^ = 0.12			
	Child birthweight (kg)	0.42	**0.02 ***	0.06	0.77
	Child breastfeeding history **^a^**	−0.46	**0.04 ***	−0.91	−0.02
	Caregiver race **^b^**	−0.11	0.69	−0.63	0.41
	Caregiver BMI	0.03	0.004 *	0.01	0.05
	Healthy food retailers	0.01	0.46	−0.02	0.04
**2**	**(Y) Child weight-for-age** **z-score (n = 115)**	Adjusted R^2^ = 0.13			
	Child birthweight (kg)	0.42	0.02 *	0.07	0.78
	Child breastfeeding history **^a^**	−0.46	0.04 *	−0.90	−0.02
	Caregiver race **^b^**	−0.11	0.68	−0.63	0.41
	Caregiver BMI	0.03	0.005 *	0.001	0.05
	Unhealthy food retailers	0.001	0.36	−0.001	0.003

All β coefficients are unstandardized. ^a^ Child breastfeeding history dichotomized as 0 = child was never breastfed and 1 = child was breastfed at least once. ^b^ Caregiver race dichotomized (0 = caregiver identified as anything besides non-Hispanic Black and 1 = caregiver is non-Hispanic Black). * Indicates statistical significance at *p* < 0.05.

**Table A4:** Food environment characteristics by SNAP participation status.

Variable	SNAP (n = 85)	Non-SNAP (n = 28)	*p*-Value
mRFEI	7.4 ± 5.6	6.1 ± 3.2	0.13
Home fruit availability	4.7 ± 3.1	7.3 ± 4.1	0.003 *
Home vegetable availability	7.4 ± 3.6	8.4 ± 3.8	0.23
Home sugary beverage availability	4.5 ± 2.7	3.9 ± 2.3	0.27
Child weight-for-age z-score	0.4 ± 1.2	0.02 ± 1.2	0.13

Independent samples *t*-tests were used to examine differences in continuous variables. * Indicates statistical significance at *p* < 0.05
